# Development of Novel Cardanol-Derived Reactive Dispersing Agents for Bio-Based Anionic–Nonionic Waterborne Polyurethane

**DOI:** 10.3390/polym16212958

**Published:** 2024-10-22

**Authors:** Jianrong Xia, Haobin Wu, Kaidong Chen, Yanling Li, Xin Lu, Sibo Ding, Xuelin Zheng

**Affiliations:** 1 Fujian Engineering and Research Center of New Chinese Lacquer Materials, Minjiang University, Fuzhou 350108, China; jrxia@mju.edu.cn; 2 College of Chemistry and Material Science, Fujian Normal University, Fuzhou 350117, China; 3 Fujian Key Laboratory of Advanced Rubber-Plastics Materials, Quanzhou 362200, China; 4 Fujian Key Laboratory of Polymer Materials, Fuzhou 350117, China

**Keywords:** cardanol, waterborne polyurethane, anionic–nonionic chain extender, photocurable, bio-based

## Abstract

This study successfully developed a bio-based, photocurable, anionic–nonionic dual-functional chain extender, and sulfonated cardanol-based polyethylene glycol (SCP), derived from renewable resources—cardanol and polyethylene glycol—for application in waterborne polyurethane dispersions (WPUDs). Utilizing SCP as a chain extender, WPUDs were prepared through a typical acetone process with poly(butylene adipate) (PBA), isophorone diisocyanate (IPDI), and ethylene diamine (EDA) at a constant NCO/OH ratio of 1:1. This research focused on the effects of polyethylene glycol molecular weight and SCP dosage on the particle size, stability, and film-forming properties of the WPUD. Optimal dispersion stability and film-forming performance were achieved with a polyethylene glycol molecular weight of 1500 and a PBA to SCP molar ratio of 4:1, yielding a particle size of 0.326 ± 0.010 μm and excellent storage stability over six months. The resulting WPU coatings exhibited a tensile strength of 11.4 MPa, which increased to 16.8 MPa after UV irradiation owing to the formation of a semi-interpenetrating network via the photopolymerization of cardanol’s unsaturated side chains. UV cross-linking also enhanced water resistance, reducing the water absorption rate (WAR) from 18.68% to 4.21% and the water vapor transmission rate (WVTR) from 6.59 × 10^−5^ g·m⁻¹·Pa⁻¹·d⁻¹ to 2.26 × 10⁻⁵ g·m⁻¹·Pa⁻¹·d⁻¹, while also improving thermal stability. These findings demonstrate that SCP offers a sustainable and effective solution for developing high-performance WPU coatings.

## 1. Introduction

Polyurethanes are indispensable in a variety of applications, such as coatings papermaking, textiles, and more, due to their versatile polymeric nature and formulation adaptability [1,2,3]. The burgeoning global environmental consciousness and tightening regulations on volatile organic compounds (VOCs) and hazardous air pollutants (HAPs) have propelled the market toward environmentally benign alternatives [4]. In this context, waterborne polyurethanes (WPUs) have emerged as a promising solution. Using water as a dispersion medium, these systems minimize or eliminate the use of organic solvents, maintaining the high performance of their solvent-based counterparts while offering additional benefits such as non-flammability, non-toxicity, reduced environmental impact, energy efficiency, and ease of storage and application [5,6,7].

Incorporating ionic groups and/or nonionic hydrophilic segments into the polyurethane backbone is an efficacious strategy for fabricating self-emulsifying WPUs [8,9,10]. Sulfonic acid-based WPUs, in particular, exhibit higher solid content [11], better mechanical properties [12], and resistance to acids, alkalis, and electrolytes compared with their carboxylic acid counterparts [13,14]. Thus, the synthesis and application of sulfonated WPUs have gained increasing attention. However, anionic emulsifiers may face limitations under low pH or high electrolyte conditions, leading to issues like phase separation and gel precipitation [15]. While nonionic hydrophilic groups improve stability against freezing, pH changes, electrolytes, and mechanical shear, they may not fully address issues related to poor storage stability and reduced water resistance [16]. Although combining anionic and nonionic emulsifiers can mitigate individual disadvantages [16,17,18,19], enhancing the mechanical properties and water resistance of WPUs often requires incorporating cross-linking agents or hybridizing with other materials [20,21,22].

In response to escalating energy and environmental challenges, the development of green and renewable resources is increasingly imperative. Biomass-derived chemicals, as exemplary renewable resources, have the potential to significantly lessen the environmental footprint of petroleum-based products [23,24,25]. Within the realm of polyurethane synthesis, polyols extracted from vegetable oils have emerged as a promising avenue [26,27,28,29]. Cardanol, a bio-oil extracted from cashew nutshells, is as an excellent natural alternative to phenols derived from petroleum [30,31]. With its unique chemical structure featuring a para-substituted unsaturated C15 alkyl chain, cardanol can undergo polymerization reactions through addition and condensation mechanisms, and various electrophilic substitution reactions including sulfonation, esterification, and nitration [30,31,32]. The abundance, cost-effectiveness, and distinctive properties of cardanol have facilitated its application across various industries, from phenolic and epoxy resins [33] to UV-curable coatings [34], surfactants [35], reactive diluents [36], and plasticizers [37], and as a polyol component in polyurethanes [38,39] and rubber composites [40]. The application of cardanol in waterborne polyurethanes has recently attracted significant interest. It is primarily utilized as a diol extender in conjunction with anionic dispersing agents for the synthesis of waterborne polyurethane emulsions [41,42] or further sulfonated to replace conventional anionic agents [43] in their synthesis. However, to the best of our knowledge, there are hardly any reports on anionic–nonionic hybrid cardanol-based dispersants for waterborne polyurethane emulsions.

This study aims to develop an anionic–nonionic reactive dispersant derived from renewable cardanol with hydrophobic unsaturated long alkyl side chains (SCP). The exploration of its application in the synthesis of WPUs is conducted, with a comparison of its performance to that of WPUs prepared using conventional industrial anionic–nonionic dispersants, such as 2-[(2-aminoethyl)amino]sulfuric acid sodium salt (AAS) and polyethylene glycol (PEG). The synthesis of SCP involves the epoxidation of cardanol, subsequent ring opening with PEG, and sulfonation. The structure of SCP was confirmed by Fourier transform infrared spectroscopy (FT-IR), nuclear magnetic resonance (NMR), and elemental analysis. This study thoroughly investigated the impact of the amount of reactive dispersant and the molecular weight of PEG within it on the stability of the WPUs and the mechanical properties of the coatings, and the effects of subsequent ultraviolet irradiation on the mechanical properties, water resistance, and water vapor transmission rate of the coatings. Given its unique attributes that combine advantages from both anionic and nonionic dispersants along with rapid cross-linking capabilities under UV irradiation, this cardanol-based reactive dispersant shows promise for developing high-performance environmentally friendly coatings that are VOC-free and surpass traditional waterborne polyurethanes.

## 2. Experimental

### 2.1. Materials

Cardanol (99.5%) was purchased from Yihuiyang New Materials Co., Ltd. (Xuzhou, China). Epichlorohydrin (ECH), triethylbenzylammonium chloride (TEBAC), triphenylphosphine (TPP), methylene chloride (CH_2_Cl_2_), chlorosulfonic acid (HClO_3_S), hydroxide (NaOH), ethylenediamine (EDA), dibutyltin dilaurate (DBTDL,), and isophorone diisocyanate (IPDI) were supplied by Aladdin Biochemical Technology Co., Ltd. (Shanghai, China). All reagents were utilized as received, without further treatment. Poly(butylene adipate) glycol (PBA, Mw = 3000 g/mol) was obtained from Aladdin (Shanghai, China), while poly(ethylene oxide glycol) (PEG, Mw = 600, 1000, 1500, 2000 and 3000 g/mol) was supplied by Sinopharm Chemical Reagent (Shanghai, China). Both PBA and PEG were degassed at 80 °C under vacuum overnight prior to use.

### 2.2. Preparation of Sulfonated Cardanol-Based Polyethylene Glycol (SCP)

**Figure 1:**
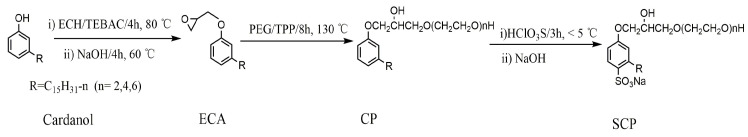
Synthetic route and schematic illustration of the molecular architecture of sulfonated cardanol-based polyethylene glycol (SCP).

The synthesis of SCP involved a two-step process—the preparation of the cardanol-based polyethylene glycol intermediate (CP) and subsequent sulfonation. Figure 1 illustrates the schematic of the synthesis processes. The cardanol-based PEG intermediate was synthesized using a method similar to those reported in the literature [43]. In this method, cardanol reacted with epichlorohydrin in the presence of triethyl benzyl ammonium chloride as a phase transfer catalyst and toluene as a solvent to produce epoxidized cardanol (ECA). Subsequently, under the catalysis of 0.5 wt% triphenylphosphine, ECA was reacted with polyethylene glycol of varying molecular weights (PEG600, PEG1000, PEG1500, PEG2000, and PEG3000) to open the epoxy ring and yield corresponding cardanol-based PEG intermediates (CP) named CP600, CP1000, CP1500, CP2000, and CP3000, respectively. For sulfonation, the cardanol-based PEG intermediate (CP, 30 mmol) was dissolved in 40 mL of dichloromethane in a 250 mL round-bottom flask and cooled to approximately 5 °C in an ice bath. With stirring, a 10 mL dichloromethane solution containing 30 mmol of chlorosulfonic acid was gradually added dropwise over 2 h. Following the sulfonation reaction, the solution pH was adjusted to neutral using a 50% sodium hydroxide solution. After filtration to remove salts, the filtrate was subjected to rotary evaporation to eliminate water and organic solvents, yielding sulfonated cardanol-based polyethylene glycols (SCP), named as SCP600, SCP1000, SCP1500, SCP2000, and SCP3000, corresponding to the molecular weight of the PEG used initially.

### 2.3. Preparation of Waterborne Polyurethane Dispersions

**Figure 2:**
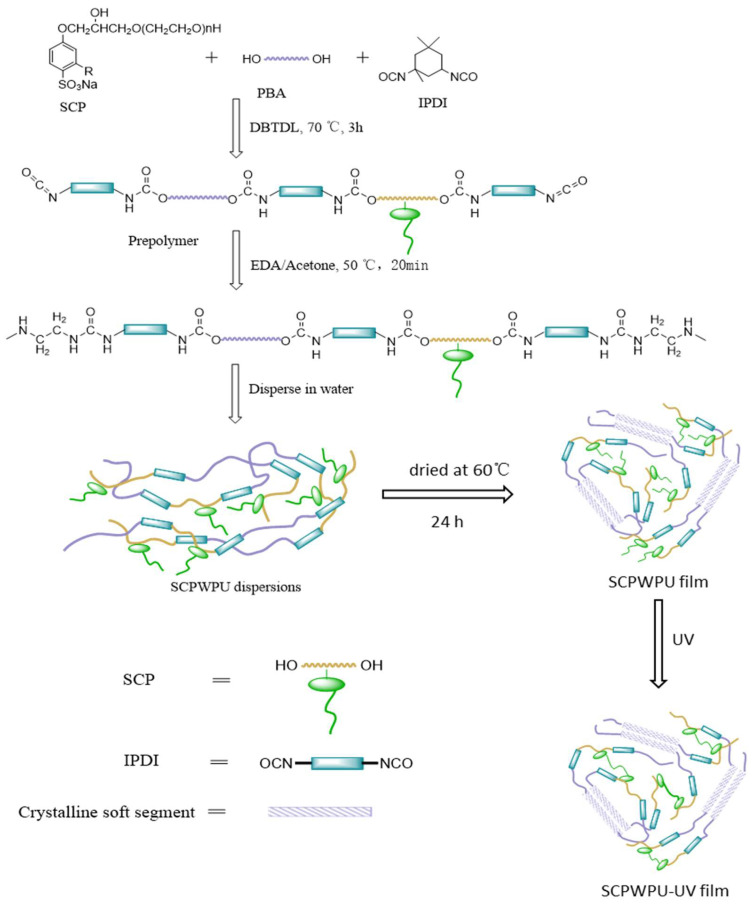
Schematic of the preparation route of SCPWPU dispersions and film.

The waterborne polyurethanes were fabricated employing the acetone process, as illustrated in Figure 2. Initially, SCP (4.25 mmol) and PBA3000 (17 mmol) were introduced into a 500 mL round-bottom flask and stirred at 60 °C under a nitrogen atmosphere until a homogeneous solution was achieved. Subsequently, IPDI (23.7 mmol) and DBTDL catalyst (0.2% by batch weight) were incorporated into the mixture and reacted at 75 °C for 3 h. EDA (3 mmol) was then added dropwise under stirring for 1 h to complete the reaction with the unreacted NCO groups. During the reaction, acetone was intermittently added to adjust viscosity while distilled water was incorporated under high shearing rates (2000 rpm) to emulsify the mixture. Finally, acetone was entirely removed by vacuum distillation. The resulting cardanol-based waterborne polyurethane dispersions were designated as SCP400WPU, SCP600WPU, SCP1000WPU, SCP1500WPU, SCP2000WPU, and SCP3000WPU, corresponding to the molecular weights of the sulfonated cardanol-based polyethylene glycols used.

For comparison, a control waterborne polyurethane dispersion (1500WPU) was prepared using PEG1500 (4.25 mmol) combined with AAS (3 mmol) as the dispersant instead of SCP, following a similar process.

### 2.4. Preparation of Waterborne Polyurethane Films

SCPWPU and WPU films were prepared by pouring the corresponding waterborne polyurethane dispersions onto polytetrafluoroethylene plates and drying at 60 °C for 24 h. Following this, the SCPWPU films were subjected to UV irradiation for further cross-linking. The films were designated based on their UV exposure time (5 s, 10 s, and 15 s) as SCPWPU-UV5, SCPWPU-UV10, and SCPWPU-UV15, respectively.

### 2.5. Characterization

The Fourier transform infrared (FTIR) spectra of all samples were obtained using an infrared spectrophotometer (Nicolet-560) in attenuated total reflectance (ATR) mode, covering a wavenumber range of 4000–400 cm^−1^. H-NMR spectra of cardanol and SCP were determined using an AVANCE II 400 NMR spectrometer (Bruker, Switzerland) at ambient temperature, with deuterated chloroform (CDCl_3_) as the solvent. Elemental analysis for sulfur content was conducted on a Vario EL cube analyzer (Elementar, Germany). Raman spectroscopy of WPU films was performed with the HORIBA (France) microscopic confocal Raman spectrometer with an excitation wavelength of 532 nm. The average particle sizes of the polyurethane dispersions were determined by a Zetasizer Nano-s (Malvern Instruments, Worcestershire, UK). Thermo-gravimetric (TG) analysis was performed with a TGA Q5000 thermogravimetric analyzer (TA Instruments, New Castle, DE, USA) under N_2_ atmosphere, with a flow rate of 30 mL/min and a heating rate of 10 °C/min. Differential scanning calorimetry (DSC) experiments were carried out on a DSC-822e instrument (Mettler Toledo) under a nitrogen atmosphere (flow rate: 40 mL/min). Samples were initially heated from 20 °C to 100 °C at a rate of 10 °C/min, followed by cooling to −30 °C and a second heating cycle to 100 °C at 2 °C/min. The first heating run was conducted to eliminate the thermal history of the samples, and the DSC curves from the first cooling and second heating cycles were recorded. The crystalline structures of all samples were investigated using X-ray powder diffraction (XRD) on a D8 ADVANCE diffractometer (Bruker, Germany) with Cu Kα radiation (λ = 0.15406 nm). Tensile properties of cured cast films were evaluated on Lloyd-LR5KPlus (Lloyd Materials Testing, West Sussex, UK) in accordance with GB/T 1040.3-2006 test standards, at a tensile rate of 50 mm/min. Seven samples from each group were measured, and outlier values were discarded using the Grubbs method. The average value and standard deviation were calculated. The water contact angle was measured by a KRÜSS contact angle measuring system, using a 5 μL water droplet as the indicator. The average of five measurements taken from different areas of the sample was calculated, with deviations reported as ±2.0°. The water absorption ratio of WPU films was evaluated by immersing the dried WPU film in deionized water at 25 °C for 24 h. Three parallel measurements were conducted for each sample. The water absorption ratio was calculated with the following equation:

Swelling (%) = (w − w_0_)/w_0_ × 100%

where w_0_ and w are the weight of dried WPU film and the film after water absorption, respectively.

The water vapor permeability (WVP) of films was measured using the desiccant method according to the standard of ASTM E96-66.

## 3. Results and Discussion

### 3.1. Origination and Structure of SCP

**Figure 3:**
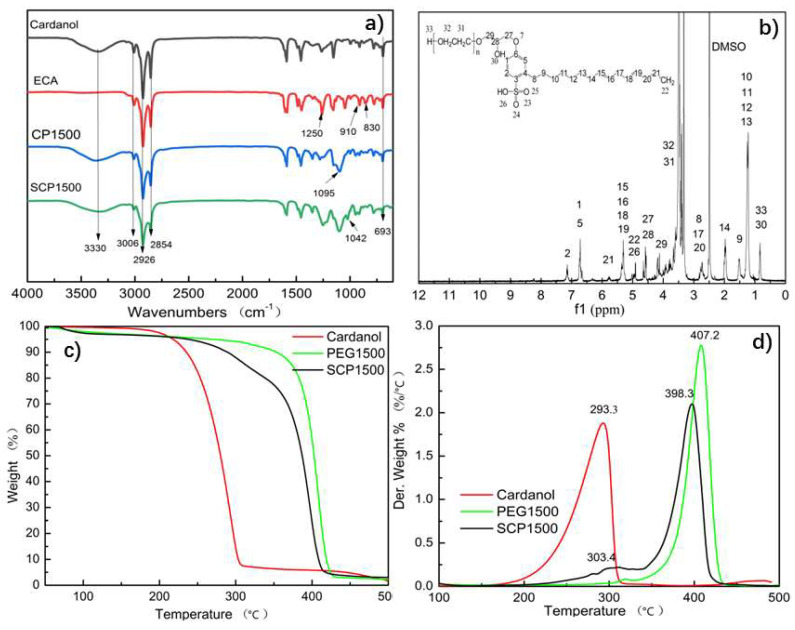
(**a**) FTIR spectra of cardanol, ECA, CP1500, and SCP1500. (**b**) ^1^H-NMR spectrum of SCP1500. (**c**) TG and DTG. (**d**) Curves of cardanol, PEG1500, and SCP1500.

The structure of the sulfonated cardanol-based polyethylene glycol (SCP) was characterized using IR spectroscopy, elemental analysis, and ^1^H-NMR. Figure 3a illustrates the IR spectra of intermediates obtained during the synthesis of SCP1500. The IR spectrum of cardanol (CA) displayed characteristic peaks corresponding to its functional groups [36,43]. A broad peak at 3342 cm^−1^ was ascribed to the O-H stretching vibration of the phenolic hydroxyl group. Peaks at 3006 cm^−1^ and 692 cm^−1^ indicated the C-H stretching and bending vibrations, respectively, associated with the unsaturated group in the side chains of cardanol. Peaks at 2926 cm^−1^ and 2854 cm^−1^ were attributed to the asymmetric stretching vibrations of the methylene and methyl groups within the alkyl side chain of cardanol. Additionally, peaks within the range of 1450–1600 cm^−1^ corresponded to the stretching vibrations of the aromatic ring in cardanol. Compared with cardanol, the absence of the characteristic peak at 3342 cm^−1^, combined with the emergence of new peaks at 1250 cm^−1^, 910 cm^−1^, and 830 cm^−1^ in the IR spectrum of epoxidized cardanol (ECA), indicated the successful introduction of the epoxide group, confirming the epoxidation reaction of cardanol’s phenolic hydroxyl group with epichlorohydrin. 

In the IR spectrum (Figure 3a) of the synthesized cardanol-based polyethylene glycol (CP1500), the characteristic epoxide group peak was absent, while the hydroxyl group peak reemerged at 3342 cm^−1^. Additionally, the C-O-C absorption band from PEG appeared at 1095 cm^−1^, confirming that a ring-opening reaction occurred between the epoxide group on ECA and the hydroxyl groups of PEG1500. In SCP1500, the presence of sulfonic acid group was indicated by a peak at 1042 cm^−1^, whereas peaks at 3006 cm^−1^ and 692 cm^−1^ related to an unsaturated C=C bond in the cardanol side chain remained intact. This suggested successful sulfonation of the cardanol-based polyethylene glycol, preserving unsaturated bonds in the long side chain.

Elemental analysis provided direct evidence of the sulfonation process of cardanol-based polyethylene glycol. As shown in Table 1, pristine cardanol, which is free of sulfur, was used as the initial material. Following sulfonation, the synthesized series of SCP (SCP600, SCP1000, SCP1500, SCP2000, SCP3000) exhibited measurable levels of sulfur consistent with theoretical values, indicating the successful incorporation of sulfonic acid groups.

The ^1^H-NMR spectrum (Figure 3b) provided further validation for the successful preparation of SCP. The characteristic peak of the sulfonic acid proton was observed at 4.94 ppm [43]. Peaks ranging from 3.7 to 3.9 ppm were attributed to the protons present in the ECH-derived segment, while the prominent peak at 3.5 ppm was indicative of the protons of PEG [44]. The hydroxyl groups were detected in the range of 0.79–0.82 ppm. Additionally, the peaks at 6.7 ppm, 6.71 ppm, and 7.13 ppm were assigned to the protons on the aromatic benzene ring of the cardanol [39]. The aliphatic protons of the side chain in cardanol [41] were represented by peaks located at 4.9 ppm, 5.28–5.73 ppm, 2.4–2.5 ppm, 1.87 ppm, 1.94 ppm, 1.48 ppm, 1.22 ppm, and 1.19 ppm.

Figure 3c,d present the thermogravimetric (TG) and differential thermogravimetric (DTG) curves for cardanol, PEG1500, and SCP1500. Cardanol underwent rapid thermal decomposition between 220 °C and 300 °C, resulting in a weight loss of 87.12 wt%, with the maximum rate of weight loss occurring at 293.3 °C. For PEG1500, the maximum weight loss occurred at a higher temperature of 407.2 °C, with a total mass loss of 91.8 wt% observed between 365 °C and 430 °C. SCP1500 exhibited two distinct stages of weight loss. The initial stage, ranging from 220 °C to 333 °C, involved a 25.3 wt% weight loss attributed to the thermal decomposition of the cardanol component. Notably, the temperature of the maximum weight loss for cardanol in SCP1500 was elevated to 303.4 °C compared with the cardanol monomer. This shift was likely due to covalent bonding between cardanol and PEG1500, which enhanced the thermal stability of the cardanol component. The second stage of weight loss for SCP1500 was observed between 365 °C and 430 °C, with a weight loss of 59.11 wt%, corresponding to the rapid thermal degradation of the PEG1500 chains. The TGA results estimated the cardanol content in SCP1500 to be 25.3 wt%, which was close to the theoretical value of 22.68 wt%. These findings, corroborated by the results of IR and ^1^H-NMR, confirmed the successful synthesis of the sulfonated cardanol-based polyethylene glycol.

### 3.2. Structure and Properties of Sulfonated Cardanol-Based Waterborne Polyurethane (SCPWPU)

**Figure 4:**
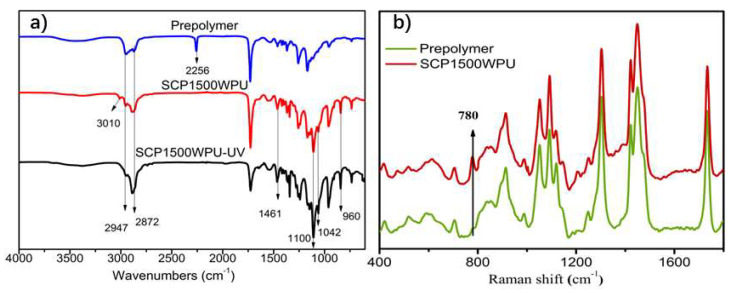
(**a**) ATR-FTIR spectra of prepolymer, SCP1500WPU, and SCP1500WPU-UV. (**b**) Raman spectra of prepolymer and SCP1500WPU.

The synthesis of waterborne polyurethane (WPU) using SCP1500 as a chain extender was monitored by FT-IR and Raman spectroscopy. The IR spectrum of the polyurethane prepolymer (Figure 4a) presented characteristic peaks at 2872–2947 cm^−1^ for -C-H stretching of -CH_2_ and -CH_3_ groups, 1729 cm^−1^ for -C=O stretching vibration, 1543 cm^−1^ for -N-H bending vibrations of the –NHCOO– group, and 2256 cm^−1^ for asymmetric -N=C=O stretching vibration [6]. For SCP1500WPU, the disappearance of the 2256 cm^−1^ peak confirmed the complete reaction of the isocyanate groups. Additionally, new peaks appeared at 1461 cm^−1^, 957 cm^−1^, and 1100 cm^−1^ corresponding to the C-O-C stretching vibrations of the PEG chain [10], and at 1042 cm^−1^ for the S-O bond. The Raman spectrum of SCP1500WPU (Figure 4b) revealed a new band at 780 cm^−1^, corresponding to the p-substituted sulfonic acid groups [45] in the cardanol benzene ring, further confirming the successful preparation of sulfonated cardanol-based waterborne polyurethane. The characteristic peak at 3010 cm^−1^ in the FT-IR spectrum indicated that the unsaturated groups [30] in the cardanol side chains still remained during WPU synthesis, suggesting the potential for further UV curing of SCPWPU films.

**Figure 5:**
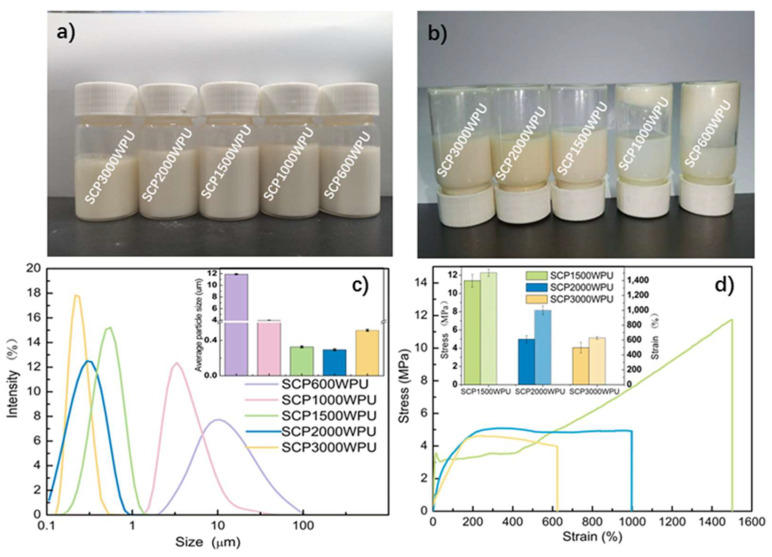
Photos of WPU emulsions prepared by SCP with different molecular weights after storage for (**a**) 1 day and (**b**) 6 months. (**c**) Particle size intensity distribution and (**d**) stress–strain curves of WPU films.

In the synthesis of WPU, hydrophilic groups play a crucial role in the emulsification process, significantly impacting the particle size and stability of the emulsion and the mechanical properties of the cured film. The influence of SCP molecular weight on the particle size of WPU dispersion was investigated using a laser particle size analyzer. As depicted in Figure 5c, the average particle sizes of SCP600WPU, SCP1000WPU, SCP1500WPU, SCP2000WPU, and SCP3000WPU dispersions were measured to be 11.90 ± 0.07 μm, 3.95 ± 0.06 μm, 0.326 ± 0.01 μm, 0.294 ± 0.01 μm, and 0.514 ± 0.01 μm, respectively. As the molecular weight of PEG in the chain extender increased, the particle size of WPU dispersion initially decreased and then increased. Lower molecular weight PEG (SCP600WPU and SCP1000WPU) introduced fewer hydrophilic groups, which limited emulsification and resulted in larger emulsion particles. After 6 months of storage, SCP600WPU and SCP1000WPU dispersions exhibited obvious precipitation and stratification (Figure 5a,b), possibly due to flocculation or agglomeration caused by strong particle attraction. In contrast, higher molecular weight PEG introduced more hydrophilic groups, improving the emulsification process and producing smaller particle sizes. The emulsion particle sizes of SCP1500WPU and SCP2000WPU were reduced to approximately 280–330 nm, with no stratification observed after 6 months of storage, indicating excellent stability. However, when the PEG molecular weight increased to 3000, the particle size of the SCP3000WPU dispersion slightly increased, likely due to the formation of a thicker hydration layer on the surface of the emulsion particles.

The mechanical properties of the cured films of SCP1500WPU, SCP2000WPU, and SCP3000WPU with excellent storage stability were further investigated. As depicted in Figure 5d, the stress–strain curves showed that the mechanical behavior of SCPWPU films varied with the molecular weight of PEG in the SCP chain extender. Specifically, the tensile strength and elongation at break of the SCPWPU films decreased from 11.4 MPa and 1505% for SCP1500WPU to 4.06 MPa and 629% for SCP3000WPU as the PEG molecular weight increased from 1500 to 3000. This observed decrease in mechanical properties was hypothesized to be related to the integration of SCP macromolecular chain extenders into the soft segments of polyurethane. Although the overall composition of the polyurethane matrix remained constant, increasing PEG molecular weight reduced the proportion of hard segments, leading to a decrease in tensile strength. Additionally, the increased viscosity and chain entanglement of PEG with higher molecular weight may hinder the mobility and extensibility of the polyurethane chains, consequently reducing the elongation at break.

**Figure 6:**
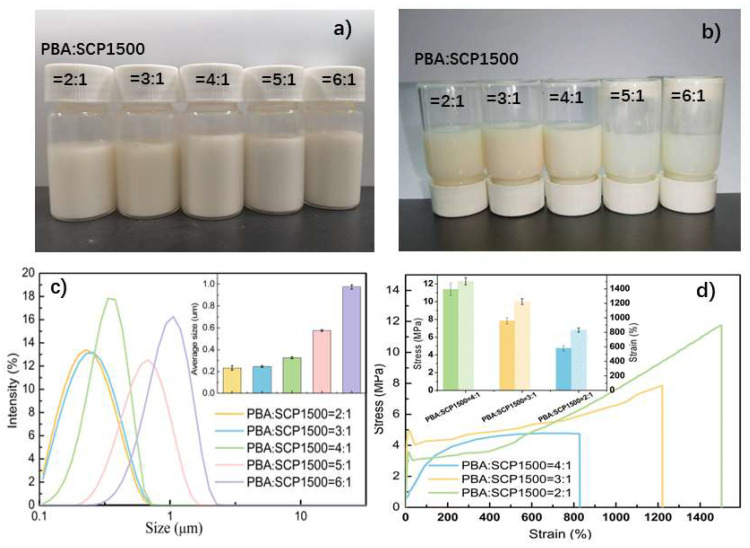
Photos of WPU emulsions prepared with different ratios of PBA/SCP1500 at (**a**) 1 day and (**b**) 6 months later. (**c**) Particle size intensity distribution and (**d**) stress–strain curves of WPU films.

Building on the optimal balance observed in SCP1500WPU, the effect of SCP1500 dosage was further investigated by varying the molar ratio of PBA to SCP1500 at a constant NCO/OH ratio of R = 1. As shown in Figure 6, reducing the PBA/SCP1500 ratio led to an increase in hydrophilic group content, which enhanced the formation of smaller emulsion particles and improved storage stability. At higher molar ratios of 6:1 and 5:1, the dispersions exhibited larger average particle sizes of 0.975 ± 0.010 μm and 0.575 ± 0.010 μm, respectively, with significant precipitation after six months of storage. In contrast, reducing the ratio to 4:1 significantly decreased the particle size to 0.326 ± 0.010 μm, and the dispersion maintained excellent storage stability over time (Figure 6b). However, while reducing the PBA/SCP1500 ratio improved storage stability, it negatively impacted the mechanical properties of the cured films. The stress–strain curves (Figure 6d) indicated that further lowing the PBA/SCP1500 ratio to 2:1 resulted in a significant reduction in both tensile strength and elongation at break.

This decline in mechanical performance can be explained by the decreased number of PBA segments, which typically exhibited stronger intermolecular forces and higher crystallinity. Additionally, the increased amount of SCP1500, with its cardanol-based flexible long side chains, likely disrupted the regularity of polyurethane matrix, weakening its structural integrity. Therefore, a PBA/SCP1500 molar ratio of 4:1 was identified as the optimal balance, providing a suitable compromise between emulsion stability and mechanical performance, making SCP1500WPU a promising candidate for practical applications.

### 3.3. Effect of UV Cross-Linking on the Performance of SCPWPU

**Figure 7:**
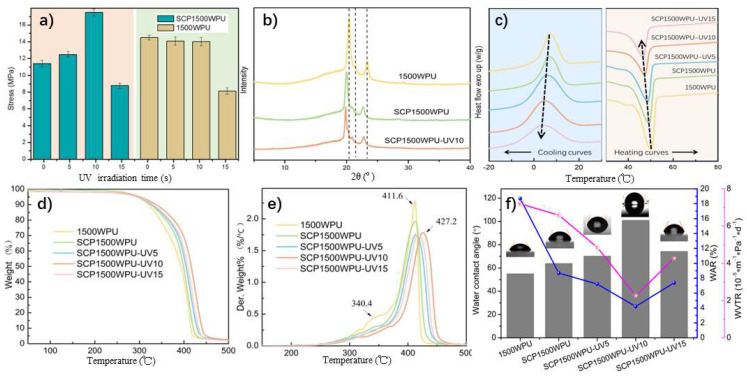
(**a**) Tensile strength of 1500WPU and SCP1500WPU films with different UV irradiation times. (**b**) XRD profiles of the 1500WPU, SCP1500WPU, and SCP1500WPU-UV10. (**c**) DSC curves of 1500WPU, SCP1500WPU, SCP1500WPU-UV5, SCP1500WPU-UV10, and SCP1500WPU-UV15 films. (**d**) TG and (**e**) DTG curves of 1500WPU, SCP1500WPU, SCP1500WPU-UV5, SCP1500WPU-UV10, and SCP1500WPU-UV15 films. (**f**) Water contact angle, water absorption rate, and water vapor transmission rate of cured films.

The incorporation of sulfonated cardanol-based polyethylene glycol (SCP1500) as a chain extender in waterborne polyurethane film enabled additional cross-linking through ultraviolet (UV) irradiation. This cross-linking was evidenced by the disappearance of the characteristic peak at 3010 cm^−1^ in the FT-IR spectrum of SCP1500WPU films after UV exposure (Figure 4a), which corresponded to the C=C bonds in the cardanol side chains. The loss of this peak indicated successful polymerization within the unsaturated long alkyl side chains of cardanol. The effect of UV irradiation on the mechanical properties of the SCP1500WPU film is illustrated in Figure 7a. Initially, the tensile strength of the SCP1500WPU film was measured at 11.4 MPa, which was lower than the 14.5 MPa observed in a structurally similar 1500WPU film prepared using a mixed nonionic–anionic chain extender (PEG1500/AAS). However, after just 10 s of UV irradiation, the tensile strength of the SCP1500WPU film significantly increased to 16.8 MPa, marking a 53.5% improvement over the non-irradiated film and surpassing the tensile strength of the 1500WPU film. In contrast, the 1500WPU film showed little to no change in its mechanical properties after 10 s of UV exposure, as depicted in Figure 7a. The observed improvement in the SCP1500WPU film suggested that, while the unsaturated long alkyl side chains of cardanol in SCP1500 may disrupt the orderly crystallization of PBA soft segments, they also impart photopolymerization capabilities upon the polyurethane film. This UV-induced photopolymerization resulted in a cross-linked structure among the SCP1500WPU molecular chains, thereby enhancing the tensile strength of the WPU film. However, upon extending the UV irradiation time to 15 s, a significant decline in the mechanical properties of both the 1500WPU and SCP1500WPU films was observed. This deterioration was likely due to the breakdown of the urethane bonds under prolonged UV irradiation, resulting in surface degradation and the formation of pores, which compromised the structural integrity of the films.

The effects of incorporating the SCP1500 chain extender and its subsequent photopolymerization on the structure of the WPU films were further investigated using X-ray diffraction (XRD) and differential scanning calorimetry (DSC). As shown in Figure 5b, the 1500WPU film displayed characteristic diffraction peaks at 2θ values of 19.2° and 23.4°, corresponding to the (120) and (112) planes of PEG segments [46], along with a peak at 21.8° attributed to the PBA (110) plane [47,48] in the polyurethane matrix. Compared with the 1500WPU film, these diffraction peaks in SCP1500WPU film showed reduced intensity and a shift to lower angles, confirming that the long aliphatic side chains from cardanol disrupted the orderly aggregation of PEG and PBA segments within the polymer to a certain extent. As a result, the crystallinity of the film decreased, and the interplanar distances increased. Moreover, after 10 s of UV exposure, the diffraction peak intensity of the SCP1500WPU film decreased even more, suggesting that the photopolymerization process created a cross-linked network that further impeded crystallization within the WPU molecular chains.

The DSC heating and cooling curves of samples are presented in Figure 7c, with detailed data summarized in Table 2. The 1500WPU film showed distinct crystallization and melting processes in the temperature ranges of 0–18 °C and 35–55 °C, with peaks for crystallization temperature (*T_c_*) at 7.7 °C and melting temperature (*T_m_*) peaks at 50.2 °C, respectively. Thermal analysis showed the enthalpy of crystallization (Δ*H_c_*) and melting (Δ*H_m_*) of 31.14 J/g and 38.31 J/g, respectively. Compared with 1500WPU film, the SCP1500WPU film exhibited a marked reduction in both crystallization enthalpy (28.68 J/g) and melting enthalpy (35.98 J/g), along with lower *T_c_* and *T_m_* peak at 6.6 °C and 49.7 °C, respectively. These reductions in enthalpy and lower crystallization and melting temperatures indicated that the long aliphatic chain of cardanol in SCP1500 disrupted the crystallization process in the soft segments of the polyurethane matrix, leading to reduced crystallinity. After 10 s of UV irradiation, the crystallization and melting peaks of the SCP1500WPU film further decreased to 4.7 °C and 47.4 °C, respectively, with the enthalpies of melting and crystallization reduced to 28.86 J/g and 23.34 J/g. This further decline in crystallinity, as indicated by the lower peak temperatures and reduced enthalpy values, was consistent with the photopolymerization of the cardanol side chains, which formed a cross-linked network that limited molecular mobility and disrupted the regular crystalline arrangement. These results aligned with the findings from the XRD analysis, corroborating the impact of photopolymerization on the film’s crystalline structure.

The thermal stability of 1500WPU film and SCP1500WPU film after various durations of UV irradiation is shown in Figure 7d. The thermogravimetric analysis (TGA) curves of all WPU films exhibited similar decomposition profiles, implying a consistent thermal decomposition mechanism across the samples. The derivative thermogravimetric (DTG) curve for the 1500WPU film (Figure 7e) showed two stages of thermal decomposition. The initial stage, ranging from 270 to 365 °C, was derived from the cleavage of urethane bonds (–COO–NH–) [10,49], while the second stage, at 365–450 °C, corresponded to the degradation of the soft segments [39], with the maximum degradation rate at 411.6 °C. The inclusion of the SCP1500 chain extender slightly enhanced the thermal stability of SCP1500WPU film compared with 1500WPU. The temperature of 50% weight loss (T_50%_) increased from 393.2 °C to 399.6 °C, likely due to the stabilizing effect of the aromatic ring structure of cardanol on the polyurethane matrix [41]. After 10 s of UV irradiation, both the T_50%_ and the temperature corresponding to the maximum weight loss rate rose to 417.2 °C and 427.2 °C, respectively. This suggested that cross-linking occurred within the unsaturated long side chains of cardanol in the SCP1500WPU film, converting linear polyurethane chains into a semi-interpenetrating network structure. The restricted mobility of molecular chains within this network contributed to the improved thermal stability of the WPU film. However, extending UV irradiation to 15 s resulted in excessive exposure, potentially causing urethane bond degradation, weaking the polyurethane backbone, and reducing thermal stability.

To further investigate the effects of the SCP1500 chain extender and UV irradiation on the hydrophobic characteristics of WPU films, water contact angle measurements were conducted using distilled water. As depicted in Figure 7f, the 1500WPU film exhibited a relatively low contact angle of 45.1°, attributed to the presence of hydrophilic groups from polyethylene glycol and ethylene diamine bis sulfonate within the film’s composition. These hydrophilic components contributed to the water-attracting nature of the surface. Incorporation of the SCP1500 chain extender, containing hydrophobic long side chains derived from cardanol, enhanced the hydrophobicity of the film, as reflected by an increased contact angle of 63.9°. After 10 s of UV irradiation, the contact angle further increased to 99.3°. This enhancement in hydrophobicity can be attributed to the cross-linking of the unsaturated long side chains in SCP1500 upon UV irradiation, which formed a more hydrophobic surface and enhanced the water-repelling properties of the WPU film.

Traditionally, WPU coatings have faced limitations due to inadequate resistance to water absorption and vapor permeation, which restricts their application in various industrial sectors [6,40]. In this study, the 1500WPU coating exhibited a relatively high water absorption rate (WAR) of 18.68% and a water vapor transmission rate (WVTR) of 7.19 × 10⁻⁵ g·m⁻¹·Pa⁻¹·d⁻¹, reflecting its susceptibility to water and moisture. The incorporation of the SCP1500 chain extender significantly enhanced the water resistance of the coating, reducing the WAR to 8.68% and the WVTR to 6.59 × 10⁻⁵ g·m⁻¹·Pa⁻¹·d⁻¹. This improvement can be attributed to the hydrophobic long side chains of cardanol within the polymer matrix, which played a crucial role in obstructing water penetration.

After 10 s of UV irradiation, the WAR and WVTR of the SCP1500WPU coating were further reduced to 4.21% and 2.26 × 10^−5^ g·m^−1^·Pa^−1^·d^−1^, respectively, surpassing the water resistance reported for most WPU films in the literature [6,12,49,50]. This enhanced water resistance was a result of the formation of a semi-interpenetrating cross-linked network via the photopolymerization of the unsaturated long side chains of cardanol. This network created an additional barrier that hindered water and moisture penetration, further enhancing the coating’s resistance. However, extending UV irradiation to 15 s led to the photodegradation of polyurethane bonds, compromising the structural integrity of the film. The appearance of micropores and bubbles on the surface significantly increased the WAR and WVTR, resulting in a notable reduction in water resistance.

## 4. Conclusions

To summarize, this research successfully synthesized a novel sulfonated cardanol-based polyethylene glycol (SCP) through a series of epoxidation, ring-opening, and sulfonation processes using cardanol and polyethylene glycol as primary materials. The structure of SCP was characterized by CHNS analysis and FT-IR and ^1^H-NMR spectroscopy. As a bio-based, environmentally friendly, and anionic–nonionic chain extender, SCP was used to produce waterborne polyurethane dispersions with photocuring properties. The effect of molecular weight of polyethylene glycol and SCP dosage on the particle size, stability, and film-forming properties of the WPU dispersions were investigated. Optimal performance was achieved with a polyethylene glycol molecular weight of 1500 and a PBA to SCP molar ratio of 4:1. This combination resulted in a WPU dispersion with a particle size of 0.326 ± 0.010 μm, demonstrating excellent storage stability over six months, and the coated film achieved a tensile strength of 11.4 MPa. The unsaturated long alkyl chains of cardanol in SCP also provided additional UV-irradiation cross-linking pathways, forming a semi-interpenetrating network that enhanced the tensile strength of the WPU coatings to 16.8 MPa after UV exposure. This cross-linking also improved the thermal stability, water resistance, and water vapor barrier properties of the coatings. This study demonstrates the potential of SCP as a sustainable high-performance chain extender for WPU dispersions, offering promising applications in environmentally friendly materials and coatings.

## Figures and Tables

**Table 1:** Sulfur content of SCP prepared with different molecular weights of PEG.

Sample	Cardanol	SCP600	SCP1000	SCP1500	SCP2000	SCP3000
Theoretical (%)	0	3.07	2.22	1.65	1.31	0.93
Practical (%)	0	2.80	1.98	1.57	1.07	0.95

**Table 2:** Thermal property of 1500WPU film and SCP1500WPU film after different durations of UV irradiation determined from DSC.

Sample	*T_c_* (°C)	Δ*H_c_* (J/g)	*T_m_* (°C)	Δ*H_m_* (J/g)
1500WPU	7.7	31.14	50.2	38.31
SCP1500WPU	6.7	28.68	49.7	35.98
SCP1500WPU-UV5	5.8	26.50	48.4	33.89
SCP1500WPU-UV10	4.7	23.34	47.4	28.86
SCP1500WPU-UV15	3.9	21.78	46.4	24.86

## Data Availability

The original contributions presented in the study are included in the article, further inquiries can be directed to the corresponding authors.
